# Mapping the regulatory landscape for environmental sustainability of medical device practices within the European Union

**DOI:** 10.1093/eurpub/ckaf262

**Published:** 2026-01-12

**Authors:** Marianna Zarro, Alessia Ferranti, Giulia Garagozzo, Giuseppe Fico, Leandro Pecchia

**Affiliations:** Departmental Faculty of Engineering, University Campus Bio-Medico, Rome, Italy; Department of Public Health and Infectious Diseases, “Sapienza” University of Rome, Rome, Italy; Departmental Faculty of Engineering, University Campus Bio-Medico, Rome, Italy; Life Supporting Technologies-LifeSTech, Universidad Politécnica de Madrid, Madrid, Spain; Life Supporting Technologies-LifeSTech, Universidad Politécnica de Madrid, Madrid, Spain; Departmental Faculty of Engineering, University Campus Bio-Medico, Rome, Italy

## Abstract

Regulatory frameworks that integrate environmental sustainability into the lifecycle of medical devices (MDs) are essential to ensure quality, safety, and effectiveness for patients while minimizing environmental impact. The Medical Device Regulation 2017/745/EC (MDR) establishes the core framework for MDs, but additional EU legislation addresses Ecodesign, sustainable packaging, financial incentives, and waste management. Although sustainability is not explicitly included in the MDR, understanding how complementary EU regulations contribute to the European Green Deal agenda is crucial to inform decisionmakers and guide future integration of sustainability principles into medical device governance. We employed a validated policy mapping methodology, derived from the scoping review approach and adapted to systematically identify and analyse regulatory documents from policy repositories rather than academic databases. This method has been previously applied in diverse policy domains, including health, education, and digital innovation. Findings were reported according to the PRISMA-ScR. Eight binding regulations that are either directly applicable or transferable to MDs in the European Union were identified. Together, they introduce requirements on Ecodesign, packaging, financial incentives, and waste management. These complement the MDR framework by embedding sustainability principles into various stages of the MD lifecycle, even though they are not explicitly mandated within the MDR itself. While environmental sustainability provisions remain absent from the MDR, complementary EU regulations create an emerging framework that supports systemic economic transformation in line with the European Green Deal. Future research should examine enforcement and practical implementation of this framework across Member States to assess its impact on medical device regulation and environmental performance.

## Introduction

In recent years, a growing body of scientific literature [1–4] has revealed a rapidly worsening ecological crisis. Anthropogenic climate change is driving an increase in average global temperatures, with associated shifts in climatic patterns observed on an annual basis. Biodiversity loss has reached critical levels, with one million of the planet’s roughly eight million species at risk of extinction, and both forests and oceans are undergoing substantial degradation due to pollution, habitat destruction, and unsustainable resource exploitation. In 2023, global greenhouse gas emissions reached 37.12 billion tonnes, and are projected to increase by 10.6% by 2030 compared to 2010 [5].

In response, the European Union has embarked on a process of modernizing and transforming its economy with the objective of climate neutrality. The regulatory framework has been reoriented towards environmental sustainability, promoting more intensive action and launching initiatives designed to promote a more circular economy and reduce the environmental impact of products throughout their entire life cycle.

The European Green Deal, presented by the European Commission in 2019, is the strategy that supports this transformation, aiming to achieve climate neutrality by 2050, in alignment with the objectives of the Paris Agreement. It aims to transform Europe into a society with a modern, resource-efficient economy that prioritises the health and well-being of its citizens [6]. One of its pillars is the Circular Economy Action Plan, adopted in 2020, which introduces measures covering the whole product life‑cycle—from design to sustainable consumption—encouraging circular economy procedures [7].

Despite these efforts, a comprehensive set of requirements ensuring that all products placed on the Union market become progressively more sustainable and compliant with circular economy principles is still lacking. European industry still follows a linear approach, as highlighted by the fact that only 12% of materials used for manufacturing new products come from recycling [6]. As a result, products are frequently replaced, entailing significant expenditure of energy and resources for manufacturing, distribution, and waste disposal [7]. This suggested that only industry mobilization across all sectors can effectively transition towards a more efficient circular economy.

Within this context, the healthcare sector plays a significant role, accounting for 4.4% of global greenhouse gas emissions [8]. It also contributes to energy consumption, and waste generation, including hazardous and single-use products. A considerable share of this environmental burden is directly linked to the production, use, and disposal of medical devices, ranging from diagnostic tools to surgical instruments and single use consumables, which rely heavily on resource-intensive manufacturing processes and generate large volumes of waste at end-of-life. As such, it represents both a challenge and an opportunity in the implementation of circular economy principles. The medical devices value chain, encompassing manufacturers, healthcare providers, distributors, and patients, engages a wide range of stakeholders, making the alignment of the sector with European Union sustainability targets not only desirable but imperative.

However, the current regulatory framework for medical devices remains primarily focused on safety and efficacy concerns. Regulation (EU) 2017/745 on medical devices (MDR) establishes safety, quality, and conformity requirements for medical devices [9]. It proposes device classification, introduces more stringent requirements for clinical evaluation, and strengthens post-market surveillance.

Although these initiatives are expected to improve safety and efficacy, the growing attention to sustainability demands closer alignment between healthcare regulation and the EU’s environmental objectives. Aligning the Medical Device Regulation (MDR) with existing EU sustainability frameworks, such as the Green Deal, the Circular Economy Action Plan, and the Ecodesign for Sustainable Products Regulation, could enhance policy coherence without duplicating regulatory burdens. Integrating environmental criteria into the MDR would not only contribute to achieving the Green Deal’s goals but also facilitate MD manufacturing adherence to environmental regulations, while stimulating innovation in the medical‑device sector, promoting the development of safer, more effective, and more sustainable technologies, also from the environmental perspective.

This article aims to provide a systematic synthesis of binding policies and regulatory approaches that promote sustainability in medical device regulation across the European Union.

## Methods

This study employed a validated policy mapping methodology, derived from the scoping review approach, and adapted to enable the systematic identification and analysis of regulatory documents from policy repositories rather than academic databases [10]. This methodology has been previously used across a range of policy domains, including health, education, and digital innovation [11–13]. Findings were reported according to the PRISMA-ScR (Preferred Reporting Items for Systematic Reviews and Meta-Analyses extension for Scoping Reviews) framework [14] and analysed through qualitative document analysis [15].

### Eligibility criteria

We examined EU-level regulatory documents related to sustainable practices for medical devices. To be eligible (Table 1), documents had to (i) be issued by governmental or competent regulatory authorities, (ii) have binding legal status, (iii) be in force between January 2015 and June 2025, and (iv) address sustainability aspects that are applicable or potentially transferable to the medical device sector. Non-binding documents such as strategies, opinions, and guidance notes were excluded. Documents published prior to 2015 but substantially amended in the study period were included in their consolidated version.

**Table 1.** Inclusion and exclusion criteria

	Inclusion criteria	Exclusion criteria
Issuing authority	Governmental or competent regulatory authorities	Other authorities
Status	Legally binding	Non-binding
Year of enforcement	2015–2025	2014 and earlier
Topic	Sustainability potentially applicable to the medical device sector	Sustainability unrelated or not transferable to the medical device sector

Given the cross-cutting nature of sustainability in medical device regulation, we also included legal texts addressing broader areas such as environmental impact, circular economy, product safety, labelling, and procurement, insofar as they explicitly related to medical technologies or devices.

### Data collection

Data collection followed a five-step process inspired by previous public health policy mapping studies. First, EU policy repository (EUR-Lex) was systematically searched using a tailored search string (available in the Supplementary Table S1). Significant keywords were selected and incorporated into the search string. These keywords were identified through an exploratory review of EU policy documents and thematic literature on medical devices and environmental sustainability, ensuring that the terms reflected the core concepts of the research question, and was validated through pilot testing to ensure comprehensive retrieval of relevant EU policy documents with acceptable specificity. In cases where combined terms yielded insufficient results, individual terms were used.

Regulatory experts were consulted, and grey literature, such as the EU Action Plan and the European Green Deal, was searched using Google to identify additional regulatory trends and reforms, with a particular focus on those linking sustainability to medical device regulation.

Third, identified documents were reviewed against eligibility criteria. All documents were independently screened by two reviewers, with discrepancies resolved through adjudication by a third reviewer. Fourth, reference lists from relevant sources were scanned to identify any additional eligible policies. Fifth, all included policies were compiled in a structured database, capturing details such as policy title, year of adoption, year of last update (if applicable), and key relevant sections.

Data collection occurred between March 2025 and July 2025.

### Data analysis

Policy documents were analysed using qualitative document analysis to extract content relevant to sustainable medical device practices. A deductive content analysis approach was used to identify and categorize recurring regulatory themes. One author performed the initial coding and thematic clustering, which was subsequently reviewed by two other authors to ensure consistency and validity.

Results were synthesized by thematic domain to enable comparison. Specifically, data were organised into five macro-areas reflecting the lifecycle phases of medical devices, as defined by Pallikarakis et al. [16], as shown in Table 2:

**Table 2.** Thematic domains used for comparative analysis of the results

Phase	Criterion
**Research and development**	Design
	Material selection
	Packaging
	Information obligations
	Economic incentives
	Voluntary and non-binding measures
**Testing and evaluation**	Data collection
	Evaluation
	Risk-benefit analysis
	Use of alternative methods
**Production and placing on the market**	Technical documentation
	Registration and traceability
	Responsibility
	Distribution and logistics
**Post-market and usage**	Performance
	Updates and maintenance
	Repair and reuse
	Management of unsold products
	Monitoring and inspections
	Sanctions
	International cooperation
**Obsolescence**	Product withdrawal
	End-of-life management
	Recovery and recycling

1. Research and Development. This phase encompasses product design, selection of materials, and packaging development. It also covers the definition of mandatory information to be included (e.g. labels, instructions), the identification of economic incentives for production, and the adoption of voluntary environmental or safety-related measures.
2. Testing and Evaluation. This phase encompasses the collection and evaluation of data related to the safety and performance of the device. It also includes risk-benefit analysis and the use of alternative testing approaches (e.g. in vitro, in silico).
3. Production and Placing on the Market. This phase encompasses the preparation of technical documentation, procedures for registration and traceability, assignment of responsibilities, and aspects related to distribution and logistics.
4. Post-Market and Usage. This phase encompasses the monitoring of the device’s performance and covers updates, maintenance, repair, and reuse practices. It also includes the management of unsold products, post-market surveillance activities, sanctions, and international cooperation frameworks.
5. Obsolescence. This phase encompasses the withdrawal of the product from the market, end-of-life management, and recovery and recycling of materials processes.

To support the practical application of these findings, we also developed a stakeholder guidance table. This table summarizes the specific obligations and roles for key stakeholder groups, including manufacturers, importers, distributors, notified bodies, and regulatory authorities, for each regulation or directive analysed. The guidance aims to clarify who is responsible for what, under each legislative framework, and highlights areas of overlap, complementarity, or potential conflict between legal instruments. For this reason, regulations and directives not yet applicable to medical devices were not included in this guidance.

## Results

We identified a total of 132 policy documents through structured searches at the European Union level. After removing duplicates and screening based on eligibility criteria, eight documents were included in the qualitative synthesis (Supplementary Table S2 reports the reasons for exclusions for the full text screening round). More detailed results are shown in the supplementary material (Supplementary Fig. S1).

The documents included a mix of binding regulations and directives requiring national implementation. These sources covered various aspects of environmental sustainability in relation to medical devices, including but not limited to design and manufacturing requirements, lifecycle and circularity principles, waste and end-of-life management, green public procurement, and reporting or labelling requirements.

As part of the thematic analysis, we conducted a detailed comparison between the MDR (2017/745/EC) and other EU legislative frameworks that are applicable to the environmental sustainability of medical devices.

The comparative analysis shows that, while the MDR provide clear guidance regarding MD safety and clinical performance, the other regulatory frameworks included in this review provide additional tools, requirements, and incentives aimed at promoting more sustainable product life cycle management. It should be noted that some of the included regulations and directives, namely Ecolabel Regulation (Ecolabel) (66/2010/EC) and Ecodesign Regulation for Electronic Displays (EED) (2019/2021/EU), are not directly applicable to the medical device sector. Nonetheless, the sustainability principles they promote could be relevant and potentially transferable to the context of medical devices. Other relevant frameworks identified in the analysis include the Waste Framework Directive (WFD) (2008/98/EC), Packaging and Packaging Waste Regulation (PPWR) (2025/40/EU), Ecodesign for Sustainable Products Regulation (ESPR) (2024/1781/EU), Waste Electrical and Electronic Equipment Directive (WEEE) (2012/19/EU) and Environmental Impact Assessment (EIA) Directive (2011/92/EU). The main findings are summarized in Table 3, following the key stages of the medical device life cycle.

**Table 3.** Characteristics of EU directives and regulations relevant to Research and Development

	MDR (2017/745/EU)	Electronic Display (2019/2021/EU)	Waste Framework (2008/98/EC)	Ecolabel (66/2010/EU)	PPWR (2025/40/EU)	EIA (2014/52/EU)	ESPR (2024/1781/EU)	WEEE (2012/19/EU)
**Design**	Safety, Performance (Annex I)	Eco-design (Art. 3)	Durability, Reparability (Art. 8)	Durability (Art. 6)	Sustainability, Recyclability (Art. 5)	–	Reusability, Durability (Art. 5)	Eco-design (Art. 4)
**Material selection**	Biocompatibility, Safety (Annex I)	Plastic, Hazardous substances restrictions (Annex II)	Recycled materials (Art. 8)	Safer alternatives(Art. 6)	Recycled, Bio-based material (Art. 7)	Substances impact (Annex IV)	Recyclability (Art. 5)	–
**Packaging**	Sterility, Integrity (Annex I)	–	Reusable packaging (Art. 9)	–	Reusability, Recyclability (Art. 1)	–	Volume and waste reduction (Annex I)	–
**Information obligations**	Safety, Performance (Annex I)	Dismantling, Spare parts (Annex II)	–	Ecolabel logo (Annex II)	Composition, Recyclability (Art. 12)	–	Performance, Environmental labels (Art. 7, 16)	Disposal, WEEE symbol (Art. 14)
**Economic incentives**	–	–	Waste prevention (Art. 4)	–	Collection, Waste prevention (Art. 26)	–	Performance classes (Art. 64)	Waste hierarchy (Art. 16 bis)
**Voluntary/non-binding**	Class I self-regulation (Art. 52)	–	Guidelines, Best practices (Art. 20)	Voluntary label (Art. 1)	–	–	Self-regulation (Art. 21)	–
**Data collection**	Data reuse, Scientific literature (Art. 61)	–	–	–	–	Environmental data (Art. 5)	–	–
**Evaluation**	Clinical evaluation (Art. 61)	–	–	–	–	Environmental evaluation (Art. 3)	–	–
**Risk–benefit analysis**	Clinical risk (Art. 62)	–	–	–	–	–	–	Health/environmental risks (Art. 5)
**Use of alternative methods**	–	–	–	–	–	Less impactful (Art. 5)	–	–
**Technical documentation**	Safety, Performance (Annex II)	Energy efficiency (Art. 4)	–	Specific criteria (Art. 9)	Sustainability compliance (Art. 39)	–	Ecodesign compliance (Annex IV)	–
**Registration & traceability**	EUDAMED, UDI (Art. 27)	–	Waste tracking (Art. 11 bis)	Registration number (Art. 9)	QR code (Art. 12)	–	Digital Passport (Art. 9)	National register (Art. 16)
**Responsibility**	Manufacturer duties (Art. 10)	Manufacturer duties (Art. 6)	Extended producer responsibility (Art. 8)	Label use (Annex IV)	Extended producer responsibility (Art. 15)	Project developer responsibility(Art. 5)	Manufacturer duties (Art. 27)	Extended producer responsibility (Art. 7)
**Distribution & logistics**	Compliance preservation (Art. 13)	–	–	–	–	–	Compliance preservation (Art. 29)	–
**Performance (Energy, etc.)**	–	Efficiency limits (Annex II)	–	–	–	Energy assessment (Annex IV)	Energy efficiency, Environmental performance (Art. 5)	–
**Updates & maintenance**	Safety-based updates (Annex I)	Software updates (Art. 6)	Waste related (Art. 9)	–	–	–	Maintainability, Upgradability (Art. 5)	–
**Repair & reuse**	Refurbishing rules (Art. 17)	Spare parts (Annex II)	Reuse (Art. 4)	Reusability (Art. 6)	Packaging reuse, Product refilling (Art. 11)	–	Repairability, Refurbishing (Art. 5)	Reuse promotion (Art. 4)
**Unsold products**	–	–	–	–	–	–	Destruction prohibition (Art. 23)	–
**Monitoring & inspections**	Safety related (Art. 86)	Eco-design related (Art. 5)	Responsibility schemes (Art. 8 bis)	Environmental performance, Compliance (Art. 10)	Reuse measures (Art. 31)	Environmental impact (Art. 8 bis)	Environmental compliance (Art. 66)	–
**Sanctions**	Safety and compliance penalties (Art. 113)	–	Violations (Art. 36)	Label misuse (Art. 17)	Sustainability (Art. 68)	National violations (Art. 10 bis)	Environmental non-compliance (Art. 74)	Legislative penalties (Art. 22)
**International cooperation**	Safety cooperation (Art. 102)	–	Responsibility schemes, Waste prevention (Art. 8)	–	–	–	–	–
**Product withdrawal**	Safety (Art. 95)	–	–	Ecolabel withdrawal (Art. 10)	Non-compliance (Art. 15)	–	Environmental non-compliance (Art. 27)	–
**End-of-Life management**	–	Dismantling (Annex II)	Waste management, Disposal (Art. 4)	–	Packaging waste plans (Art. 42)	–	–	E-waste collection (Art. 5)
**Recovery & recycling**	–	Design for recycling (Annex II)	Separate collection, quantitative targets (Art. 4)	–	Recycling targets (Art. 52)	–	Design for recycling (Art. 5)	Collection & Recovery targets (Art. 7)

The rest of this section presents a structured synthesis of the findings, organized according to the five lifecycle phases defined in the method section.

### Research and development

Regarding the research and development phase, the WFD, Ecolabel, PPWR, ESPR, WEEE and EED directly promote sustainable considerations, explicitly encouraging design strategies aimed at enhancing durability, reparability, and waste reduction. In particular, the ESPR introduces specific eco-design requirements that address premature obsolescence by establishing specific durability criteria.

A key aspect influencing product sustainability during the design phase is material selection. While the MDR only prioritizes biocompatibility and patient safety, particularly concerning carcinogenic, mutagenic and reprotoxic (CMR) substances and endocrine disruptors, the EED, WFD, PPWR, Ecolabel, and ESPR emphasize the reduction of these substances for both health and environmental reasons.

Moreover, the PPWR and ESPR introduce specific environmental requirements for packaging, including reductions in weight and volume, as well as the use of reusable and recyclable materials. The MDR addresses packaging only from the perspective of ensuring device sterility and integrity, without considering its environmental impact.

Information obligations also differ significantly. While the MDR provides information to ensure a safe use of medical devices, the EED, PPWR, ESPR, and WEEE require mandatory environmental information (e.g. waste management, recyclability) and the use of environmental labels such as the WEEE symbol and ESPR performance classes.

Finally, the WFD, PPWR, ESPR, and WEEE also include economic instruments and incentives to support the prevention of waste and the development of more sustainable products.

### Testing and evaluation

Regarding the testing and evaluation phase, the MDR focuses on collecting and assessing clinical data to ensure the safety and performance of medical devices. It promotes the reuse of existing data and scientific literature to reduce the need for new testing and requires risk-benefit analyses with particular attention to the safety of human subjects. Moreover, the MDR requires mandatory clinical evaluation and risk-benefit assessments of medical devices. In contrast, the other regulatory frameworks provide limited consideration of this phase. Among them, the EIA requires the collection of environmental data and an environmental impact assessment as a requirement for project approval. The ESPR considers environmental risks in relation to sustainability benefits, while the WEEE focuses on health and environmental risks associated with waste management.

Regarding the promotion of alternative testing methods, while the MDR does not explicitly promote the use of sustainable methods such as *in vitro* and in silico approaches, the Ecolabel includes requirements to reduce animal testing.

### Production and placing on the market

Regarding the production and placing on the market phase, the MDR requires manufacturers to prepare technical documentation demonstrating compliance with safety and performance requirements. Similarly, the PPWR and the ESPR mandate technical documentation proving compliance with sustainability criteria related to packaging and ecodesign, respectively.

Most of the regulatory frameworks include registration and traceability mechanisms, but with different scopes. The MDR establishes a robust system through the European database on medical devices (EUDAMED) and the Unique Device Identifier (UDI), enabling traceability from development to usage. The PPWR introduces QR codes and producer registries, while the ESPR implements a Digital Product Passport to ensure traceability throughout the entire life cycle. The WFD and WEEE require producer registration and reporting obligations concerning waste flows, and Ecolabel assigns registration numbers to certified products.

Regarding the responsibility, the MDR assigns primary responsibility to the manufacturer, while importers and distributors are responsible for ensuring compliance during transport and storage. The WEE, PPWR, and WFD introduce the principle of Extended Producer Responsibility (EPR), which includes the accountability for waste management, following the “polluter pays” principle.

Finally, distribution and logistics are explicitly addressed in the MDR and ESPR, focusing on maintaining product safety and integrity during transport and storage.

### Post-market and usage

Regarding the post-market and usage phase, the MDR establishes requirements to ensure device performance and safety without requiring energy efficiency. It only demands that the energy state of the device be determined. In contrast, the ESPR introduces obligations on energy and environmental performance during the usage phase and the Ecolabel energy efficiency among the criteria for product certification.

While the MDR promotes software updates and maintenance to ensure safety and performance, the ESPR and the WFD encourage maintainability and upgradability to avoid premature obsolescence and reduce waste production, respectively.

Regarding repair and reuse, the MDR allows for national-level regulation on refurbishing and establishes requirements for spare parts availability solely concerning device safety. The EED, WFD, Ecolabel, PPWR, ESPR, and WEEE explicitly promote repairability, reuse, and refurbishment. The PPWR, for example, sets obligations for the reuse of packaging and systems for the collection of reusable packaging.

Among the documents analysed, only the ESPR addresses the management of unsold products and prohibits their destruction, imposing annual transparency and reporting obligations.

All the regulatory frameworks include post-market surveillance mechanisms and sanctions for non-compliance, but with different scopes. The MDR establishes a post-market surveillance system focused on incidents and safety monitoring, requiring regular reporting obligations. The ESPR introduces a market surveillance system, including data collection on energy consumption and environmental performance. The EIA requires a description of monitoring measures for environmental impacts as a requirement for project approval.

Concerning sanctions, the MDR delegates the definition of penalties to Member States and limits their scope to safety-related violations, while the ESPR and PPWR explicitly introduce sanctions for non-compliance with environmental obligations.

### Obsolescence

Regarding the obsolescence phase, the MDR mandates product withdrawal when devices present risks to health and safety. By comparison, the PPWR and ESPR establish procedures for product and packaging withdrawal in case of non-compliance with environmental requirements. While the Ecolabel mandates the revocation of the certification in case of non-compliance.

With regard to end-of-life management, the MDR does not include considerations for the treatment or disposal of devices at the end of their lifecycle, as well as their recovery or recycling. However, Article 14 of Annex I requires that devices be designed and manufactured to enable their safe disposal. Manufacturers are therefore expected to identify and test appropriate disposal procedures, which must be clearly described in the instructions for use. In contrast, the WFD, PPWR, and WEEE impose obligations to ensure safe waste management, including the definition of management plans and annual reporting requirements.

Regarding recovery and recycling, the WFD and WEEE promote material recovery and recycling, setting quantitative targets and encouraging separate collection systems.

The review revealed that environmental sustainability considerations in medical device regulation across Europe are primarily addressed through product lifecycle requirements, circular economy principles, digitalization for sustainability tracking and end-of-life management. Some degree of heterogeneity was observed in the depth and specificity of environmental sustainability provisions, especially when comparing EU-level legislation with national-level implementation.

It is important to note that EU legislation applies across Member States unless superseded by stricter national provisions or, in the case of non-EU countries such as the UK, only up to the date of formal withdrawal from EU legal obligations. In countries without national transposition of relevant EU legislation, EU directives may still apply directly depending on the legal framework in place.

## Discussion

Environmental sustainability in medical device regulation is addressed through multiple EU legal instruments, both binding and requiring national transposition. Five lifecycle phases (from R&D to obsolescence) were used to organize the analysis, highlighting how sustainability requirements vary across the lifecycle. A stakeholder guidance tool was developed to support the practical application of these findings (available in the Supplementary Data). It may be particularly useful in the context of large-scale European projects involving the testing of innovative technologies, where navigating ethical and regulatory requirements across multiple jurisdictions remains a major challenge, as highlighted by Maccaro et al. in the GATEKEEPER project [17].

The study confirms the increasing relevance of environmental sustainability in Medtech regulation, as anticipated by EU Green Deal and Circular Economy Action Plan. However, numerous studies have emphasized the need for a stronger integration of sustainability principles within the medical device sector [8, 18], particularly in relation to digital health [19, 20]. While digital health is increasingly promoted as a strategic pillar in national and European health agendas, environmental sustainability remains an underrepresented objective in most initiatives, including funding programs [18]. Unlike previous reviews that focused primarily on Life Cycle Assessment (LCA) methodologies [21] or industrial sustainability standards [8, 22, 23], this study provides an overview of regulation instruments. The results highlight the integration of sustainability goals into traditionally safety-focused regulatory frameworks like the MDR.

Building on these findings, several regulatory and policy enhancements can be envisioned to advance environmental sustainability in the MedTech sector. In fact, we did not collect evidence regarding the enforcement and practical implementation by Member States. National-level initiatives may be underrepresented or show divergence due to the absence of harmonized reporting mechanisms. Moreover, potential publication bias may favour well-documented EU-wide policies over more fragmented or less visible national practices.

The current MDR could be further strengthened by systematically integrating sustainability principles into its framework. This includes the selection of materials based not only on biocompatibility and safety for human health but also on criteria aimed at minimizing environmental impact across the device life cycle. Packaging requirements, for example, could evolve to encompass parameters such as weight, volume, and the use of reusable or recyclable materials, alongside the existing standards for sterility and safety. Additionally, incorporating environmental information into product labelling could enhance transparency and support informed decision-making among stakeholders. In this context, manufacturers engaging in research and development could adopt a ‘safe and sustainable by design’ framework, similarly to what has been suggested for the pharmaceutical sector [24], to ensure that environmental sustainability considerations are integrated from the early stages of product development, without compromising patient safety and while maintaining the core regulatory objective of protecting human health throughout the device life cycle [25].

This need for alignment between environmental sustainability and health objectives is not limited to the European level. At the global level, ongoing negotiations for a Global Plastics Treaty aim to reduce plastic pollution, yet currently proposed drafts include broad exemptions for medical products, including medical plastics [26]. At the global level, this misalignment is also reflected in the limited scope of existing international standards. While ISO 14001 and ISO 14040/14044 provide general frameworks for environmental management and life cycle assessment, they are not specific to medical devices [27–30]. IEC 60601-1-9 introduces some sustainability requirements for medical electrical equipment, but its coverage is narrow [8], and the ISO 10993 series touches sustainability only indirectly through material considerations [31, 32].

Economic incentives may play a critical role in facilitating the transition towards more sustainable medical devices. Such incentives could support manufacturers in developing eco-friendly alternatives and adopting waste prevention strategies. The systematic collection of environmental data, together with structured environmental impact assessments, would provide an evidence base for evaluating both environmental risks and potential sustainability benefits associated with specific devices or production processes. In this regard, incorporating environmental sustainability dimensions within Health Technology Assessment (HTA) processes could further strengthen evidence-informed decision-making by enabling a more comprehensive appraisal of the long-term environmental externalities associated with novel medical technologies [33, 34], even though such integration remains a limited practice to date [35]. Moreover, the wider adoption of Green Public Procurement (GPP) practices, by systematically integrating environmental sustainability criteria into public tenders, could further stimulate market demand for greener medical technologies, although its implementation remains uneven and not yet fully consolidated in routine procurement processes [36, 37].

Sustainability criteria, particularly those related to packaging and eco-design, could be formally included within the scope of the technical documentation required for conformity assessment. The adoption of Extended Producer Responsibility (EPR), in alignment with the “polluter pays” principle, would ensure that manufacturers are held accountable for the environmental consequences of their products throughout their life cycle, including disposal and waste management.

In addition, manufacturers could play a key role in advancing circular economy practices by implementing take-back systems and developing product-as-a-service systems [38], which may significantly reduce waste generation and extend product life cycles by shifting business models from product ownership to service-oriented delivery.

To further align with circular economy principles, the regulatory framework should promote design for repairability, reuse, and refurbishment. Finally, market surveillance activities could be broadened to monitor not only the safety and performance of medical devices but also their energy consumption and overall environmental footprint.

The stakeholder guidance tool developed as part of this study could help operationalize these recommendations, particularly by supporting stakeholders in the early integration of sustainability criteria into conformity assessment, procurement planning, and regulatory strategy.

Future research should include empirical investigations into how manufacturers and national competent authorities operationalize sustainability-related obligations under existing and emerging regulatory frameworks. There is also a need to assess the real-world impact of such regulations on medical device design, production costs, and market availability, in order to identify potential trade-offs and implementation challenges. Additionally, the development and validation of harmonized indicators to monitor sustainability compliance and enforcement outcomes at the national level would support more consistent evaluation and benchmarking across Member States. Systematic analyses of ISO and other international standards in the context of medical device applications and environmental sustainability are also warranted, to identify gaps, overlaps, and opportunities for alignment with regulatory requirements. Finally, future research should also explore how sustainability regulations for medical devices may affect patient access to care and patient outcomes, to better understand potential implications for clinical practice and healthcare delivery.

The EU is gradually embedding sustainability into MedTech regulation. Greater alignment between environmental and health regulatory and policy goals is needed for coherent implementation. The stakeholder guidance tool developed in this study may support more effective planning and collaboration, particularly among small and medium-sized manufacturers, procurement agencies, and regulators. In the long term, integrating sustainability into clinical and policy decision-making could enhance environmental resilience while maintain high standards of patient safety and outcomes.

## Supplementary Material

ckaf262_Supplementary_Data

## Data Availability

All analysed documents are publicly accessible through EU-level policy repositories. No new datasets were generated. As the data used are publicly available and concern existing legal frameworks, no ethical approval or consent procedures were required. Key points Regulatory frameworks complementing the Medical Device Regulation (MDR) embed sustainability principles across the medical device lifecycle, supporting quality, safety, and environmental impact reduction. EU legislation on Ecodesign, sustainable packaging, financial incentives, and waste management reinforces the European Green Deal agenda in Medtech regulation. Future research should assess enforcement, practical implementation, and real-world impact of sustainability provisions across Member States.
