# Supplementary material for: Mapping the regulatory landscape for environmental sustainability of medical device practices within the European Union
Source: Eur J Public Health. 2026 Jan 12;36(2):ckaf262. doi: 10.1093/eurpub/ckaf262 (PMC13064526; doi:10.1093/eurpub/ckaf262)
Supplement: ckaf262_Supplementary_Data [file ckaf262_supplementary_data.zip › ejph-2025-09-om-0834-File002.pdf]

Supplementary data 1. Stakeholder guidance tool

Phases					
Regulation	MDR				
Stakeholder	Manufacturer	Importer	Authorised Representative	Distributor	Healthcare institution
**Research and Development** (Design, Material Selection, Packaging, Information Obligations, Economic Incentives, Voluntary Measures)	Ensure that devices are designed and manufactured in conformity with the requirements of this Regulation when they are placed on the market or put into service (Art. 10.1).<br>Establish, document, implement and maintain a risk-management system in accordance with Annex I, point 3 (Art. 10.2).<br>Establish, document, implement, maintain, update and improve a quality-management system proportionate to the risk class and type of device, ensuring compliance with the Regulation (Art. 10.9).<br>Hold financial coverage proportionate to the risk class, type of device and size of the undertaking, with regard to potential liability for damage caused by a defective device (Art. 10.16).				
**Testing and Evaluation** (Data Collection, Evaluation, Risk-Benefit Analysis, Use of Alternative Methods)	Carry out a clinical evaluation in accordance with Art. 61 and Annex XIV, including PMCF (Post-Market Clinical Follow-up) (Art. 10.3).<br>Plan, conduct and document, under Art. 61 and Annex XIV, Part A, a clinical evaluation based on sufficient clinical data (including, where relevant, Annex III data) demonstrating compliance with the general safety, performance and benefit-risk requirements of Annex I, points 1 and 8, in relation to the device's characteristics and intended purpose (Art. 61.1).<br>For class III devices and class IIb devices (Art. 54.1(b)), an				
**Production and Placing on the Market** (Technical Documentation, Registration and Traceability, Responsibility, Distribution and Logistics)	Draw up and keep up to date the technical documentation (Annexes II and III) for devices other than custom-made devices, in order to allow their conformity assessment (Art. 10.4).<br>For custom-made devices, draw up, update and make available to the competent authorities the documentation required by Annex XIII, point 2 (Art. 10.5).<br>Draw up the EU declaration of conformity (Art. 19) and affix the CE marking after demonstrating the conformity of devices (other than custom-made or investigational devices) by means of the appropriate conformity-assessment procedure (Art. 10.6).<br>Continuously update the EU declaration of conformity attesting compliance with the Regulation, containing at least the information in Annex IV and translated into the languages of the countries where the device is made available (Art. 19.1).<br>Keep the technical documentation, the EU declaration of conformity and, where applicable, a copy of the certificate (issued under Art. 56) for at least 10 years after the last device covered by the declaration has been placed on the market (15	Place on the Union market only devices that comply with this Regulation (Art. 13.1).<br>Before placing on the market, verify that:<br>• the CE marking has been affixed and the EU declaration of conformity has been drawn up (Art. 13.2.a)<br>• the manufacturer is identified and has appointed an authorised representative in accordance with Article 11 (Art. 13.2.b)<br>• the device is labelled in accordance with the Regulation and is accompanied by the required instructions for use (Art. 13.2.c)<br>• the manufacturer, where applicable, has assigned a UDI to the device in accordance with Article 27 (Art. 13.2.d).<br>Refrain from placing on the market a device considered non-compliant until it has been brought into conformity, informing the manufacturer and the authorised representative. If the device is believed to present a serious risk or is falsified, also inform the competent authority (Art. 13.2, last paragraph).<br>Indicate on the device or its packaging or in an accompanying document your name, trade mark or trade name, registered	Verify that the EU declaration of conformity and the technical documentation have been drawn up and, where applicable, that the manufacturer has carried out an appropriate conformity-assessment procedure (Art. 11.3.a).<br>Fulfil the registration obligations laid down in Art. 31 and verify that the manufacturer has complied with the registration obligations under Arts 27 and 29 (Art. 11.3.c).<br>Carry out the tasks specified in the mandate concluded with the manufacturer and, on request, provide a copy to the competent authority (Art. 11.3).<br>Register in Eudamed before placing a non-custom-made device on the market, uploading (if not already registered) the information in Annex VI, Part A, point 1; where conformity assessment involves a notified body, upload those data before submitting the application to that body (Art. 31.1).<br>Record and keep, preferably in electronic form, the UDIs of devices supplied or received that fall under class III implantables or any additional devices/categories/groups designated by an act	Make a device available on the market only in accordance with the applicable requirements (Art. 14.1).<br>Before making a device available on the market, verify that:<br>• the CE marking has been affixed and the EU declaration of conformity has been drawn up (Art. 14.2.a)<br>• the device is accompanied by the information required by the manufacturer under Article 10.11 (Art. 14.2.b)<br>• for imported devices, the importer has fulfilled the requirements of Article 13.3 (Art. 14.2.c)<br>• the manufacturer, where applicable, has assigned a UDI to the device (Art. 14.2.d).<br>Refrain from making available a device considered non-compliant until it has been brought into conformity, informing the manufacturer and, where	Record and keep, preferably in electronic form, the UDIs of class III implantable devices supplied or received; for other devices, record and keep the UDIs if this is required or encouraged by national legislation (Art. 27.9)
**Post-Market and Usage** (Performance, Updates and maintenance, Repair and reuse, Management of unsold products, Monitoring and inspections, Sanctions, International Cooperation)	Set up and keep up to date the post-market surveillance system in accordance with Art. 83 (Art. 10.10).<br>Plan, establish, document, implement, maintain and update a post-market surveillance system proportionate to the risk class and type of device, integrating it into the quality-management system (Art. 83.1).<br>For class I devices, draw up a post-market surveillance report summarising the results and conclusions of the data analysis, reasons and any preventive/corrective actions taken; update it when necessary and provide it to the competent authority on request (Art. 85).<br>For each class IIa, IIb and III device, draw up a periodic safety update report (PSUR) summarising the results and conclusions of post-market surveillance, the benefit-risk conclusions, the main PMCF findings and sales/usage data; update it at least annually (classes IIb and III) or every three years (class IIa) and include it in the technical documentation. Submit the PSUR via Eudamed to the notified body for class III or implantable devices;	Keep a register of complaints, non-compliant devices, recalls and withdrawals, and provide the manufacturer, authorised representative and distributors with the information needed to examine complaints (Art. 13.6).<br>If a device placed on the market is found to be non-compliant, immediately inform the manufacturer and the authorised representative, cooperate in corrective actions and, in the event of a serious risk, notify the competent authorities and the notified body (Art. 13.7).<br>Forward promptly to the manufacturer and authorised representative complaints and reports of incidents received from healthcare professionals, patients or users (Art. 13.8).<br>Cooperate, on request, with the competent authorities in any action taken to eliminate or mitigate the risks posed by devices that you have placed on the market; also supply free samples or ensure access to the device (Art. 13.10).<br>Cooperate with manufacturers/authorised representatives to achieve an appropriate level of traceability of devices (Art. 25.1).	Provide the competent authorities, upon request, with all information and documentation needed to demonstrate the device's conformity, in the language required by the Member State concerned (Art. 11.3.d).<br>Forward to the manufacturer any request from a Member-State competent authority for samples or access to devices, ensuring that the authority actually receives the samples or gains access (Art. 11.3.e).<br>Immediately inform the manufacturer of complaints and reports from healthcare professionals, patients or users concerning suspected incidents related to the devices covered by the mandate (Art. 11.3.g).<br>Terminate the mandate if the manufacturer breaches the obligations of the Regulation, immediately informing the competent authority and, where applicable, the notified body involved in the device's conformity assessment, stating the reasons for termination (Arts 11.3.h & 11.6).<br>Be liable for defective devices where the manufacturer (without	If a device already made available on the market is found to be non-compliant, immediately inform the manufacturer and, where applicable, the authorised representative and the importer, cooperating with them on corrective actions. If the device is considered to present a serious risk, also inform the competent authority, providing details of the non-compliance and the measures taken (Art. 14.4).<br>Transmit promptly to the manufacturer and, where applicable, to the authorised representative and the importer, any complaints and reports of incidents relating to a device; keep a register of complaints, non-compliant devices, recalls and withdrawals, keeping those parties informed of such monitoring (Art. 14.5).<br>Upon request of a competent authority, provide all information and documentation necessary to	Make available to all patients in whom the device has been implanted the information defined in Article 18.1, by any means that allows rapid access (Art. 18.2)
**Obsolesence** (Product withdrawal, End-of-life management, Recovery and recycling)	Keep the technical documentation, the EU declaration of conformity and, where applicable, a copy of the certificate (issued under Art. 56) for at least 10 years after the last device covered by the declaration has been placed on the market (15 years for implantable devices) (Art. 10.8).<br>Be able to provide the competent authority, for the entire retention period laid down in Art. 10.8 (10/15 years), with the identification of:<br>• every economic operator to whom the device was supplied directly;<br>• every economic operator that supplied the device directly;<br>• every healthcare institution or healthcare professional to whom the device was supplied directly (Art. 25.2).	Keep, for at least 10 years (15 years for implantable devices) after the last device has been placed on the market, a copy of the EU declaration of conformity and, where applicable, the certificate issued under Article 56, including any amendments and supplements (Art. 13.9).<br>Be able to provide the competent authority, for the entire retention period laid down in Art. 10.8 (10/15 years), with the identification of:<br>• every economic operator to whom the device was supplied directly;<br>• every economic operator that supplied the device directly;<br>• every healthcare institution or healthcare professional to whom the device was supplied directly (Art. 25.2).	Keep at the disposal of the competent authorities, for the period set out in Art. 10.8 (10 years / 15 years for implantable devices), a copy of the technical documentation, the EU declaration of conformity and, where applicable, the relevant certificate issued under Art. 56, including any amendments (Art. 11.3.b).<br>Be able to provide the competent authority, for the entire retention period laid down in Art. 10.8 (10/15 years), with the identification of:<br>• every economic operator to whom the device was supplied directly;<br>• every economic operator that supplied the device directly;<br>• every healthcare institution or healthcare professional to whom the device was supplied directly (Art. 25.2).	Be able to provide the competent authority, for the entire retention period laid down in Art. 10.8 (10/15 years), with the identification of:<br>• every economic operator to whom the device was supplied directly;<br>• every economic operator that supplied the device directly;<br>• every healthcare institution or healthcare professional to whom the device was supplied directly (Art. 25.2).	

Waste Framework		Ecolabel		PPWR	
**Manufacturer**	**Waste management operator**	**Manufacturer**	**Importer**	**Manufacturer**	**Importer**
The person who first uses a material that has ceased to be waste must ensure that the material complies with the legislation on chemicals and products before such legislation becomes applicable to that material. (Art. 6.5)		Submit an application to the competent body for use of the label, selecting the body according to the product's origin (Art. 9.1)<br>State in the application full contact details, the relevant product group, a detailed description of the product, and any additional information requested by the competent body (Art. 9.3)<br>Provide the missing documentation within 6 months of the notice of incompleteness, otherwise the application will be rejected (Art. 9.5)<br>Ensure that all production sites comply with any technical requirements laid down in the Ecolabel criteria (Art. 9.6)<br>Affix the EU Ecolabel and its registration number to the product only after signing the contract with the competent body (Art. 9.9)<br>Use the label solely on products for which it has been awarded and on related promotional material; misleading use is prohibited (Art. 9.11; 10.1)	Submit an application to the competent body for use of the label, selecting the body according to the product's origin (Art. 9.1)<br>State in the application full contact details, the relevant product group, a detailed description of the product, and any additional information requested by the competent body (Art. 9.3)<br>Provide the missing documentation within 6 months of the notice of incompleteness, otherwise the application will be rejected (Art. 9.5)<br>Ensure that all production sites comply with any technical requirements laid down in the Ecolabel criteria (Art. 9.6)<br>Affix the EU Ecolabel and its registration number to the product only after signing the contract with the competent body (Art. 9.9)<br>Use the label solely on products for which it has been awarded and on related promotional material; misleading use is prohibited (Art. 9.11; 10.1)	Manufacture packaging so as to minimise the formation of micro-plastics during waste management (Art 5.1)<br>Packaging (and their components) shall not contain substances restricted by Annex I or by other EU legislation above the permitted limits (Art 5.2)<br>Assess the recyclability of packaging and classify it in classes A,B or C as per Table 3 of Annex II (Art 6.3)<br>Label packaging (Art 12)<br>Reduce empty space: by 12 February 2028 for sales packaging; by 1 January 2030 (or 3 years after implementing act) for multipack, transport, and e-commerce packaging (max 50% empty space) (Art. 24).<br>Provide consumers with information on waste prevention, reuse options, separate collection, the impact of improper disposal, and the compostability of packaging; from 12 August 2028, also include the meaning of labels and symbols (Art. 55.1)	
Producers of hazardous waste must keep a chronological register containing information on the quantity, nature, origin, destination, and treatment of the waste, as well as on the materials recovered. These data must be made available to the authorities through electronic registers. (Art. 35.1)<br>Registers relating to hazardous waste must be kept for at least three years. (Art. 35.2)		Authorise access to production premises when requested by the competent body; the request may be made at any reasonable time, even without prior notice (Art. 10.4)	Authorise access to production premises when requested by the competent body; the request may be made at any reasonable time, even without prior notice (Art. 10.4)	Place on the market only packaging compliant with Articles 5-12 (Art 15.1)<br>Before placing on the market, carry out the conformity assessment in Annex VII and issue the EU declaration of conformity (Art 15.2)<br>Keep the technical documentation and the EU declaration for 5 years (single-use packaging) or 10 years (reusable packaging) (Art. 15.3)<br>Continuously ensure production conformity and repeat assessment when design, characteristics or standards change (Art 15.4)<br>Mark each packaging with an identifier (type, batch or serial number) or include it in accompanying documents if marking on pack is impossible (Art 15.5)<br>Indicate on the packaging or via QR-code the manufacturer's name and, where available, electronic contact details (Art 15.6)<br>Ensure that the information referred to in Article 15.5-6 is	Place on the market only packaging that complies with the requirements of Articles 5–12 (Art 18.1)<br>Before placing on the market, verify that the manufacturer has carried out the conformity assessment and drawn up the EU declaration; if not compliant, do not place on the market (Art 18.2)<br>Indicate on the packaging or label your name/trade mark and address so it is legible, indelible and does not obscure other mandatory information (Art 18.3-4)<br>Ensure that storage or transport under your responsibility does not compromise packaging conformity (Art 18.5)<br>Keep the technical documentation and the EU declaration for 5 years (single-use packaging) or 10 years (reusable packaging) (Art 18.7)<br><br>Label packaging (Art 12)<br>From 1 January 2030, do not place on the market the packaging formats and uses listed in Annex V. (Art. 22.1)
Producers of hazardous waste are subject to regular inspections by the competent authorities. (Art. 34.1)		Allow the competent body to carry out any investigations needed to monitor continuous compliance with the Ecolabel criteria and the obligations under Art. 9 (Art. 10.3)	Allow the competent body to carry out any investigations needed to monitor continuous compliance with the Ecolabel criteria and the obligations under Art. 9 (Art. 10.3)	If non-compliant packaging is detected, immediately undertake corrective measures and inform the competent authority (Art 15.8)<br>Provide the authorities within 10 days of the request with documentation proving compliance (Art 15.10)<br>Upon request, must provide market surveillance authorities with the identity of both suppliers and recipients of packaging or packaged products, and must retain this information for 5 years in the case of single-use packaging and 10 years for reusable packaging (Art. 22).<br>Participate in reuse systems for reusable packaging and ensure that those systems comply with Annex VI, part A. (Art. 27.1);<br>Recondition reusable packaging in accordance with Annex VI, Part B (Art. 27.2)<br>Optionally designate third parties to operate common reuse systems (Art 27.3)<br>In closed-loop systems compliant with Annex VI, return	If packaging is suspected non-compliant, undertake corrective measures and inform market-surveillance authorities (Art 18.6)<br>Provide authorities within 10 days of request all information needed to prove conformity (Art 18.8)<br>Co-operate with competent authorities in any action aimed at correcting non-compliance (Art 18.9)<br>Upon request, must provide market surveillance authorities with the identity of both suppliers and recipients of packaging or packaged products, and must retain this information for 5 years in the case of single-use packaging and 10 years for reusable packaging (Art. 22).<br>Participate in reuse systems for reusable packaging and ensure that those systems comply with Annex VI, part A. (Art. 27.1);<br>Recondition reusable packaging in accordance with Annex VI, Part B (Art. 27.2)<br>Optionally designate third parties to operate common reuse
The costs of waste management, including those related to the necessary infrastructure and its operation, shall be borne by the producer. (Art. 14.1)<br>Transferring waste to another party for preliminary treatment does not automatically relieve the producer of responsibility for its final recovery or disposal. (Art. 15.2)	Entities involved in waste management are subject to regular inspections by the competent authorities. (Art. 34.1)<br>Companies and entities managing hazardous waste must keep a chronological register containing information on the quantity, nature, origin, destination, and treatment of the waste, as well as on the recovered materials. These data must be made available to the authorities through electronic registers. (Art. 35.1)<br>Registers relating to hazardous waste must be kept for at least three years. (Art. 35.2)			Take corrective measures or withdraw any non-compliant packaging placed on the EU market (Art 15.8-58.4).	Take corrective measures or withdraw any non-compliant packaging placed on the EU market (Articles 18.6-58.4).

		EIA	ESPR			
**Distributor**	**Waste management operator**	**Project developer**	**Manufacturer**	**Importer**	**Distributors**	**Manufacturer**
		Submit to the competent authority all the minimum information required by Annex IV (project description, alternatives, mitigation measures, non-technical summary, etc.) (Art. 5.1)<br>Describe measures for monitoring significant environmental impacts of the project (Art. 8a)	Design and manufacture the device in compliance with the performance requirements (Art. 27.1.a)<br>Attach all environmental information required by the Articles 4-7 (Art. 27.1.b)			Design the device in line with eco-design principles so that it can be easily dismantled, reused and recycled; avoid solutions that hinder recovery unless they are justified by safety or environmental-protection requirements (Art. 4).<br>Finance the collection, treatment, recovery and environmentally sound disposal of household WEEE arising from EEE placed on the market after the relevant cut-off dates (Art. 12.1 a-c).<br>Bear the costs of WEEE stemming from their own products, individually or via a collective system, and provide a financial guarantee covering future WEEE management when the product is placed on the market (Art. 12.3).<br>Finance the collection, treatment, recovery and environmentally sound disposal of WEEE
		Evaluate all significant impacts as a condition for project authorization (Art. 3, Annex III).<br>Collect environmental data and use existing studies to assess project impact (Art. 5, Annex IV).				
Make available on the market only compliant packaging and exercise due diligence with respect to the Regulation's requirements (Art 19.1)<br>Before making packaging available, check that the upstream obligations are fulfilled and required markings are present; if not, withhold it until conformity is achieved (Art 19.2-3)<br>Use the information received from the producer only to verify conformity; any commercial misuse is forbidden (Art 19.4)<br>Ensure that storage and transport under your control do not compromise conformity (Art 19.3)<br><br>Label packaging (Art 12)<br>From 1 January 2030, do not place on the market the packaging formats and uses listed in Annex V. (Art. 22.1)<br>Reduce empty space: by 12 February 2028 for sales packaging; by 1 January 2030 (or 3 years after implementing act) for multipack, transport, and e-commerce packaging (max 50% empty space) (Art. 24).			Make the product's digital passport available and keep it up to date; ensure it conforms with the articles 4-9 (Art. 27.1.c)<br>Perform the conformity assessment and draw up the technical documentation before placing on the market any product covered by a delegated act (Art. 27.2)<br>If the product is compliant, draw up an EU declaration of conformity and affix the CE marking (or an alternative mark) (Art. 27.2)<br>Keep the technical documentation and the EU declaration of conformity for 10 years from the date the product is placed on the market (Art. 27.3)<br>Ensure that series production remains compliant; repeat the assessment if the product or standards change (Art. 27.4)<br>Affix a batch/series number or other element that allows identification of the product (Art. 27.5)	Place on the market only products that meet the requirements set out in the delegated acts (Art. 29.1)<br>Before placing a product on the market, verify that the manufacturer has carried out the conformity assessment and drawn up the technical documentation, ensure that the product bears the required labels and digital product passport, verify that the manufacturer's digital product passport, including a backup copy, is available. Do not place a product on the market if you suspect it is not in conformity with the requirements laid down in the delegated acts (Art. 29.2)<br>Indicate on the product (or in the digital passport) your name, trade name, postal and electronic address so you can be contacted (Art. 29.3)	Before placing a product on the market, verify that the product bears the CE mark (or alternative) and that the manufacturer has fulfilled the obligations of Articles 27.5-6 and 29.3 (Art. 30.2.c)<br>If doubt arises about the product's conformity or manufacturer's compliance, do not place it on the market until the matter is resolved; ensure that storage and transport under your responsibility do not compromise conformity (Art. 30.3)<br>Guarantee customers (including distance sales) access to the labels and digital product passport specified in the delegated acts that accompany the product (Art. 31.1)<br>Ensure that the digital product passport is easily accessible to customers and potential customers, including online (Art. 31.2)	Transmit, free of charge, to national authorities the data on WEEE that they (or third parties acting on their behalf) have collected separately (Art. 7.2.c).<br>Keep documentation on the weights of WEEE, components, materials or substances:<br>– leaving collection facilities (output);<br>– entering and leaving treatment facilities (input/output);<br>– entering recovery/recycling/preparation-for-reuse facilities (input);<br>– and on the weights of products or materials leaving recovery/recycling/preparation facilities (Art. 11.4-6).
If you suspect packaging is non-compliant, stop making it available and immediately inform the authorities (Art 19.5)<br>Provide the national authority, on justified request, all information proving conformity and cooperate with corrective action (Art 19.6)<br>Upon request, must provide market surveillance authorities with the identity of both suppliers and recipients of packaging or packaged products, and must retain this information for 5 years in the case of single-use packaging and 10 years for reusable packaging (Art. 22).<br>Participate in reuse systems for reusable packaging and ensure that those systems comply with Annex VI, part A. (Art. 27.1);<br>Recondition reusable packaging in accordance with Annex VI, Part B (Art. 27.2)<br>Optionally designate third parties to operate common reuse systems (Art 27.3)<br>In closed-loop systems compliant with Annex VI, return the			Immediately take corrective actions and inform the authorities if the product is found to be non-compliant (Art. 27.8)<br>Provide public communication channels (phone number, email, or web section) to allow submission of product complaints (Art. 27.9)<br>Provide any document requested by the authorities within 15 days to demonstrate conformity (Art. 27.10)<br>Co-operate with market-surveillance authorities; remove or counter offers of non-compliant products (Art. 35)<br>Provide free of charge the information available or access to data required by the delegated act for conformity verification (Art. 38)<br>It is forbidden to circumvent ecodesign requirements or use software that alters performance during tests (Art. 40)	If the product is found to be non-compliant: take corrective measures or inform the competent authority (Art. 29.6)<br>Keep a copy of the EU declaration of conformity and make it available to the authorities for 10 years after the product is placed on the market (Art. 29.7)<br>Upon reasoned request from the authority, provide all the information demonstrating conformity within 15 days and co-operate with any corrective measures (Art. 29.8)<br>Co-operate with market-surveillance authorities; remove or counter offers of non-compliant products (Art. 35)<br>Provide free of charge the information available or access to data required by the delegated act for conformity verification (Art. 38)	If a product already distributed is found non-compliant: take corrective actions and immediately inform the market-surveillance authorities (Art. 30.4)<br>Provide, upon reasoned request of the authority, all information and documentation showing conformity within 15 days and co-operate with any corrective action (Art. 30.5)<br>Co-operate with market-surveillance authorities; remove or counter offers of non-compliant products (Art. 35)<br>Provide free of charge the information available or access to data required by the delegated act for conformity verification (Art. 38)<br>It is forbidden to circumvent ecodesign requirements or use software that alters	Organise and manage, individually and/or collectively, take-back systems for WEEE (waste electrical and electronic equipment) from private households, ensuring the directive's targets are met (Art. 5.2.d).<br>Arrange the collection of WEEE from users other than private households (Art. 5.5).<br>Contribute, through extended-producer-responsibility schemes, to achieving the minimum collection rate set by the Member State: at least 65 % of the average weight of EEE (electrical and electronic equipment) placed on the market (Art. 7.1).<br>Monitor target attainment using the prescribed calculation method (weight of WEEE entering recovery/recycling facilities ÷ weight of all separately collected WEEE in the same category). Preliminary operations (sorting,
Take corrective measures or withdraw any non-compliant packaging placed on the EU market (Art 19.5-58.4).	Transmit annually to the competent authorities the data on packaging waste listed in Table 3 of Annex XII (excluding packaging placed on the market for the first time), using one or more electronic registers (Art. 23)<br>Transmit annually to producers (or their EPR schemes) all data needed for them to comply with the information duties in article 44, paragraph 10 (Art 23)		Immediately take corrective actions or withdraw and inform the authorities if the product is found to be non-compliant (Art. 27.8)	If the product is found to be non-compliant: take corrective measures or withdraw) or inform the competent authority (Art. 29.6)	If a product already distributed is found non-compliant: take corrective actions or withdraw and immediately inform the market-surveillance authorities (Art. 30.4)	Establish, individually or collectively, systems for recovering WEEE, using the best available techniques (Art. 8.3).<br>Achieve the minimum recovery, recycling and preparation-for-reuse rates for each WEEE category as laid down in Annex V (Art. 11.1).

WEEE	
Distributor	Waste management operator
Provide, if required by the Member State, the information in Article 14.2-4 (e.g. meaning of the symbol, obligation of separate collection) in the instructions for use, at points of sale or through awareness-raising campaigns (Art. 14.5).	
Transmit free of charge to the Member State the information on the quantities of WEEE received in their sales outlets or warehouses (Art. 7.2.b).	Transmit, free of charge, to national authorities the data on WEEE received at collection or treatment facilities (Art. 7.2.a).
Ensure, at the time a new product is supplied, the free "one-for-one" take-back of an equivalent WEEE that has performed the same functions (Art. 5.2.b).<br>In retail outlets with an EEE sales area ≥ 400 m² (or in their immediate vicinity), carry out the free collection of very small WEEE (< 25 cm) with no purchase obligation for the final user (Art. 5.2.c).	
	It is prohibited to dispose of separately collected WEEE before it has undergone the treatments provided for in Article 8 (Art. 6.1).<br>Collect and transport separately collected WEEE in a manner that ensures optimal conditions for preparation for reuse, recycling or containment of hazardous substances (Art. 6.2).<br>Treat all separately collected WEEE appropriately, including at least: removal of all liquids and selective treatment in accordance with Annex VII (Art. 8.1-2).<br>Store and treat WEEE in compliance with the technical requirements of Annex VIII (Art. 8.3).

Note:
Yellow-shaded boxes mark requirements addressed first to the Member State, not directly to the actor listed in the column. It is therefore up to each Member State to transpose the rule and set up the legal, administrative or enforcement mechanisms that compel the actor to comply in practice.
