# Supplementary material for: Mapping the regulatory landscape for environmental sustainability of medical device practices within the European Union
Source: Eur J Public Health. 2026 Jan 12;36(2):ckaf262. doi: 10.1093/eurpub/ckaf262 (PMC13064526; doi:10.1093/eurpub/ckaf262)
Supplement: ckaf262_Supplementary_Data [file ckaf262_supplementary_data.zip › ejph-2025-09-om-0834-File003.pdf]

Supplementary data 2. Thematic analysis of included documents.

		MDR	Electronic Display (2019/2021/EU)	Waste Framework (2008/98/EC)	Ecolabel (66/2010/EU)	PPWR (2025/40/EU)	EIA (2014/52/EU)	ESPR (2024/1781/EU)	WEEE (2012/19/EU)
**General**	Objectives and Purpose	Ensures the safety and performance of medical devices throughout their active lifecycle (Art. 1).	Promotes the eco-design of electronic displays (Art. 1).	Defines measures to prevent or reduce the negative impacts of waste production and management (Art. 1).	Establishes the EU Ecolabel scheme (Art. 1).	It focuses on packaging sustainability, contributing to the reduction of packaging waste and promoting the circular economy (Art. 1).	Ensures environmental protection by assessing the significant environmental impacts of a project before its implementation (Art. 1).	Ensures eco-design to promote durability, repairability, reusability, and recyclability of products (Art. 1, 3).	Ensures the protection of the environment and human health by preventing and reducing negative impacts from WEEE (Art. 1).
	Responsible Body	European Commission, MDCG (Medical Device Coordination Group), national competent authorities (Art. 103–105), and notified bodies for certification (Art. 35–50).	European Commission, competent national authorities.	European Commission, competent authorities, European Environment Agency (Art. 11bis), and cooperation with the European Chemicals Agency (Art. 9).	European Commission, European Union Ecolabelling Board (EUEB), national competent bodies (Arts. 4, 5).	European Commission, competent national authorities (Art. 40), European Environment Agency (Art. 41)	National environmental authorities designated by the Member States (Art. 6).	European Commission (Art. 4), industry stakeholders, and standardization bodies. The Ecodesign Forum acts as an advisory body (Art. 19–20).	European Commission, national environmental authorities, producers through collective systems (Art. 16–18), authorized operators for WEEE collection (Art. 5).
	Scope of Application	Medical devices and accessories during their active lifecycle, and clinical investigations (Art. 1, 2)	Electronic displays, but excludes displays intended for medical use (Art. 1).	All types of waste (Art. 2).	Goods and services supplied for distribution, consumption, or use. Excludes medicinal products and medical devices (Art. 2).	It applies to packaging and packaging waste, with requirements covering the entire life cycle of packaging (Art. 2).	Public and private projects likely to have significant environmental impacts (Art. 1, 4, Annexes I–II), applied during the pre-authorization phase.	Any physical good placed on the EU market (Art. 1), including medical devices, without compromising the health and safety of patients and users.	AEE, including medical devices, but explicitly excludes infected in vitro diagnostic medical devices before end-of-life, active implantable medical devices, or equipment intended for research and development purposes (Art. 2).

Supplementary data 2. Thematic analysis of included documents.

		**MDR** (2017/745/EU)	**Electronic Display** (2019/2021/EU)	**Waste Framework** (2008/98/EC)	**Ecolabel** (66/2010/EU)	**PPWR** (2025/40/EU)	**EIA** (2014/52/EU)	**ESPR** (2024/1781/EU)	**WEEE** (2012/19/EU)
**Research and Development**	Design	Requirements focus on safety and clinical performance (Annex I).	Provides eco-design specifications, for example regarding energy efficiency (Art. 3; Annex II).	Promotes design for durability, reparability, and waste reduction (Arts. 8, 9, Annex IV)	Environemntal criteria promote durability (Art. 6, Annex I)	Sustainability requirements such as minimum recycled content in plastic packaging, bio-based raw materials, and reduction of packaging weight and volume (Arts. 5–11, 23). Recyclability performance classes for packaging (Annex II).	Not provided.	Establishes design requirements for reusability, repairability, and maintenance (Art. 5, Annex I). Avoids premature obsolescence with specific durability requirements.	Promotes the application of eco-design principles (Art. 4).
	Material Selection	Based on biocompatibility and patient safety, with specific attention to CMR substances and endocrine disruptors (Annex I).	Requires marking of plastic components weighing more than 50g, restrictions on hazardous substances, and information on the presence of flame retardants (Annex II).	Encourages the use of recycled materials and the reduction of hazardous substances (Arts. 8, 9).	Environemntal criteria promote the substitution of hazardous substances with safer alternatives (Art. 6)	Restrictions on the use of hazardous substances and heavy metals in packaging (Art. 5). Promotion of recycled and bio-based materials (Arts. 7, 8).	Requires assessment of the impact of substances used in the project (Annex IV).	Based on reducing substances of concern, improving recycled content, recyclability, and recovery potential (Art. 5).	Not provided.
	Packaging	Designed to ensure sterility and integrity of the device (Annex I).	Not provided.	Supports the use of reusable packaging (Art. 9).	Not provided.	General and specific design requirements for packaging, including environmental impact minimization, reusability, and recyclability.	Not provided.	Incorporates principles of Directive 94/62/EC, defining design requirements to reduce packaging volume and minimize packaging waste (Annex I).	Not provided.
	Information Obligations (labeling, technical sheets, etc.)	Labels and instructions are intended to ensure identification, safe use, and clinical performance (Annex I).	Obligation to provide information on dismantling, repair instructions, and availability of spare parts (Annex II).	Not explicitly required, but encourages the labelling of hazardous waste (Art. 19).	Label with the Ecolabel logo (Annex II)	Mandatory labelling with information on composition, recyclability, and waste management (Art. 12, Annex VII); consumer information on waste prevention (Art. 55).	Not provided.	Information obligations regarding performance, installation, use, maintenance, collection, and treatment (Art. 7). Environmental labels with performance classes (Art. 16,17).	Obligation to include the WEEE symbol on products and provide consumer information on proper disposal, environmental risks, and collection systems (Art. 14, Annex IX).
	Economic Incentives (design support)	Not provided.	Not provided.	Promotes economic instruments to support waste prevention and the waste hierarchy (Art. 4, Annex IV, IVbis).	Not provided, but the use of the Ecolabel is promoted through awareness-raising and dissemination activities (Art. 12)	Incentives for collection, waste prevention, and packaging reuse (Arts. 26, 43, 51). Green public procurement, defining mandatory minimum requirements to support sustainability (Art. 63).	Not provided.	Economic incentives for products with top environmental performance classes (Art. 64). Promotes green public procurement by defining minimum technical specifications, award criteria, and minimum targets to support sustainability (Art. 65).	Economic incentives to promote the application of the waste hierarchy (Art. 16 bis).
	Voluntary and Non-binding Measures (self-regulation, guidelines, best practices)	Class I: self-regulation without notified bodies; manufacturers may independently assess conformity, draft the EU declaration, and affix CE marking (Art. 52).	Not provided.	Provides guidelines for cross-border cooperation and for the collection of hazardous household waste (Art. 20).<br>Encourages voluntary instruments (e.g., EMAS, ISO 14001), best practices, and sectoral agreements (Annex IV).	It is a voluntary environmental certification label (Art. 1)	Not provided.	Not provided.	Allows economic operators to propose alternative design requirements (self-regulatory measures) (Art. 21, Annex VI).	Not provided.

Supplementary data 2. Thematic analysis of included documents.

		MDR	Electronic Display (2019/2021/EU)	Waste Framework (2008/98/EC)	Ecolabel (66/2010/EU)	PPWR (2025/40/EU)	EIA (2014/52/EU)	ESPR (2024/1781/EU)	WEEE (2012/19/EU)
**Testing and Evaluation**	Data Collection (clinical or environmental)	Promotes the reuse of existing data and scientific literature to reduce the need for new testing when appropriate (Art. 61).	Not provided.	Not provided.	Not provided, but the Ecolabel criteria are based on life cycle data and quantitative environmental impacts (Annex I).	Not provided.	Requires collecting environmental data and using existing studies to assess project impact (Art. 5, Annex IV).	Not provided.	Not provided.
	Evaluation (clinical and environmental)*	Requires clinical evaluation to demonstrate safety and effectiveness (Art. 61). Regulates clinical investigations on human subjects, focusing on safety and dignity (Art. 62, 64–68).	Not provided.	Not provided.	Non provided.	Not provided.	Imposes environmental evaluation covering all significant impacts as a condition for project authorization (Art. 3, Annex III).	Not provided.	Not provided.
	Risk–Benefit Analysis	Requires clinical risk–benefit analysis with protection of participants in testing (Art. 62, 64–68, Annex I).	Not provided.	Not provided.	Not regulated, but Ecolabel criteria consider the net environmental risk-benefit balance (Art. 6, Annex I).	Not provided.	Requires impact evaluation, but does not formalize a comparative risk–benefit analysis	Considers environmental risks in relation to the product's sustainability benefits, particularly for medical devices.	Evaluates health and environmental risks associated with waste collection and treatment in relation to benefits (Art. 5, 8).
	Use of Alternative Methods	Does not promote sustainable alternative methods (e.g., in silico, in vitro models).	Not provided.	Not provided.	Not regulated, but the Ecolabel criteria take into account the principle of reducing animal testing (Art. 6).	Not provided.	Implicitly encourages the use of less impactful alternatives when available (Art. 5, Annex IV).	Not provided.	Not provided.

* valutazione clinica: processo atto a produrre, raccogliere, analizzare e valutare in maniera continuativa i dati clinici relativi a un dispositivo per verificarne la sicurezza e le prestazioni, benefici clinici compresi

** valutazione conformità: verifica che i requisiti normativi siano soddisfatti

Supplementary data 2. Thematic analysis of included documents.

		**MDR**	**Electronic Display** (2019/2021/EU)	**Waste Framework** (2008/98/EC)	**Ecolabel** (66/2010/EU)	**PPWR** (2025/40/EU)	**EIA** (2014/52/EU)	**ESPR** (2024/1781/EU)	**WEEE** (2012/19/EU)
**Production and Placing on the Market**	Technical Documentation	Requires technical documentation demonstrating the device's compliance with safety and performance requirements (Annexes II and III).	Technical documentation must include measured technical parameters for energy efficiency and justification for the absence of marking on plastic parts (Art. 4, Annex VI of Regulation 2019/2013).	Not provided.	Requires an application documentation according to the specific criteria for each product group (Art. 9).	Detailed technical documentation demonstrating compliance with sustainability requirements (Art. 39, Annex VII).	Not provided.	Requires technical documentation to demonstrate compliance with ecodesign requirements (Annex IV).	Not provided.
	Registration and Traceability	Requires EUDAMED registration and a UDI system for identification and traceability (Arts. 27–33).	Not provided.	Waste tracking system and mandatory authorisation/registration for operators (Arts. 11bis, 22–26, 35–36).	Registration number system for certified products (Art. 9).	Traceability systems through QR codes on packaging along the value chain to ensure verification of sustainability credentials (Art. 12). Traceability system for packaging waste (Art. 53). Harmonized databases on packaging and packaging waste flows (Art. 57).	Not provided.	Mandatory Digital Product Passport with unique identifiers and traceability throughout the entire life cycle (Arts. 9–14).	Manufacturers' registration in national registers. Annual reporting on quantities placed on the market, collected, and recycled (Art. 16).
	Responsibility	Primary responsibility lies with the manufacturer. Importers and distributors are responsible for transport, storage, and verification of compliance (Arts. 10, 13,14).	Manufacturers and importers are responsible for ensuring that products do not circumvent assessments and comply with eco-design requirements (Art. 6).	Extended producer responsibility for waste management (Arts. 8, 8bis).<br>"Polluter pays" principle regarding waste costs and management responsibilities (Arts. 14–15).	Responsibility for the way the Ecolabel is used (Annex IV).	Extended producer responsibility scheme covering packaging waste management costs, including labelling and monitoring (Arts. 15, 45–47). Importers, distributors, and suppliers' responsibility for transport and storage (Arts. 18, 19, 20). Producer register (Art. 44).	The project developer is responsible for the accuracy of environmental information and the implementation of mitigation measures (Art. 5,7).	Primary responsibility lies with the manufacturer. Importers and distributors are responsible for transport and storage (Arts. 27,29,30,76).	Extended producer responsibility for the collection, treatment, and financing of end-of-life WEEE (Arts. 7,12,13).
	Distribution and Logistics	Importers and distributors must ensure appropriate transport and storage conditions to preserve the device's compliance (Arts. 13,14).	Not provided.	Not provided.	Not provided.	Not directly addressed, but it sets packaging design requirements that ensure safety during transport and logistics (Annex IV).	Not provided.	Importers and distributors must ensure appropriate transport and storage conditions to preserve the device's compliance (Arts. 29,30).	Not provided.

Supplementary data 2. Thematic analysis of included documents.

		MDR	Electronic Display (2019/2021/EU)	Waste Framework (2008/98/EC)	Ecolabel (66/2010/EU)	PPWR (2025/40/EU)	EIA (2014/52/EU)	ESPR (2024/1781/EU)	WEEE (2012/19/EU)
**Post-Market and Usage**	Performance (consumption, energy efficiency)	Does not regulate energy efficiency; requires the energy status of the device to be determinable (Annex I).	Sets energy efficiency limits in on-mode and power consumption limits in standby and off modes (Annex II)	Not provided.	Not regulated, but Ecolabel criteria consider environmental performance (Annex I).	Not provided.	Requires assessment of energy use in the project design (Annex IV).	Introduces requirements for energy efficiency and environmental performance during the operational phase (Art. 5, 6, Annex I). Contributes to the objectives of Directive 2018/2002/EU.	Not provided.
	Updates and maintenance	Considers maintenance and updates only if necessary for the safety and functionality of the device (Annex I).	Obligation to ensure that software updates do not degrade the environmental performance of the product and to provide maintenance support (Art. 6, Annex II).	Upgradability promoted to reduce waste (Art. 9).	Not provided.	Not provided.	Not provided.	Promotes maintainability and upgradability of products, avoiding planned obsolescence (Art. 5, Annex I).	Not provided.
	Repair and Reuse	Refurbishing is allowed only if regulated at national level, especially for single-use devices (Art. 17, Annex I). Regulates spare parts without compromising safety (Art. 23).	Obligation to make essential spare parts available for at least 7 years and to ensure they can be replaced with commonly available tools without permanent damage to the appliance (Annex II).	Promotion of reuse and preparation for reuse (Arts. 4, 9, 11).	Ecolabel criteria promote reusability (Art. 6, Annex I).	Requirements for the reuse and refurbishment of packaging and for product refilling, in compliance with health and safety (Arts. 11, 27, 28, Annex VI). Obligations for the collection of reusable packaging through dedicated systems (Arts. 26, 27, 31) and minimum reuse targets for specific categories (Art. 29).	Not provided.	Promotes repairability, spare parts availability, and reuse; encourages refurbishing (Art. 5, Annex I).	Promotes eco-design to facilitate reuse (Art. 4).
	Management of unsold products	Not provided.	Not provided.	Not provided.	Not provided.	Not provided.	Not provided.	Prohibits destruction of unsold products and imposes transparency obligations on their management (Art. 23–26). Requires annual publication of information on unsold products (Art. 24).	Not directly applicable, but unsold products that become waste fall under the general obligations for collection and treatment provided by the directive.
	Monitoring and inspections	Post-market surveillance focused on incidents and safety (Art. 83–100, Annex III). Periodic safety reports are mandatory for manufacturers (Art. 86).	Provides for market surveillance procedures to verify compliance with eco-design requirements (Art. 5).	Monitoring system for extended producer responsibility schemes (Art. 8bis). Inspections and enforcement by competent authorities (Art. 34).	Monitoring of environmental performance and checks on compliance with Ecolabel criteria (Art. 10).	European observatory on reuse to monitor implementation of reuse measures (Art. 31). The monitoring of compliance is entrusted to the competent national authorities (Arts. 40–41).	Requires description of measures for monitoring significant environmental impacts of the project (Art. 8bis)	Introduces a market surveillance system to verify compliance with environmental requirements (Art. 66–69). Introduces the obligation to monitor and report product data (Art. 37), such as quantities placed on the market, energy consumption, and environmental parameters.	Not provided for the use phase but includes obligations for monitoring of the directive's implementation (Art. 23).
	Sanctions	Provides for national penalties for violations related to safety and compliance (Art. 113).	Not provided.	Provides for penalties in case of violations (Art. 36).	Penalties for misuse of the Ecolabel (Art. 17).	Sanction regime for violations of sustainability requirements (Art. 68).	Requires Member States to establish penalties for violations of national provisions adopted under the directive (Art. 10bis).	Provides for nationally defined penalties for environmental non-compliance (Art. 74).	Provides for nationally defined penalties for violations of the legislation (Art. 22).*
	International Cooperation	Cooperation limited to safety and market surveillance (Art. 102).	Not provided.	Cross-border cooperation on producer responsibility schemes, market surveillance, and waste prevention/management programmes (Arts. 8, 32, 38).	Not provided.	Not provided.	Not provided.	Not provided.	Not provided for the use phase but promotes information exchange among competent authorities to monitor the implementation of the legislation (Art. 18).

Supplementary data 2. Thematic analysis of included documents.

		MDR	Electronic Display (2019/2021/EU)	Waste Framework (2008/98/EC)	Ecolabel (66/2010/EU)	PPWR (2025/40/EU)	EIA (2014/52/EU)	ESPR (2024/1781/EU)	WEEE (2012/19/EU)
**Obsolesence**	Product Withdrawal	Mandatory withdrawal procedures for devices that pose health and safety risks (Art. 95).	Not provided.	Not provided.	Suspension or withdrawal of the right to use the Ecolabel (Art. 10, Annex IV).	Withdrawal procedures for packaging that does not comply with the regulation (Arts. 15, 18, 19, 58–63).	Not provided.	Withdrawal procedures for products that do not comply with environmental requirements (Art. 27,29,30,69).	Not provided.
	End-of-Life Management	Not provided.	Requires that design does not prevent dismantling of components (Annex II).	Regulates waste management and disposal at end-of-life, ensuring protection of human health and the environment (Arts. 4, 12, 13). Establishes waste management plans (Art. 28).	Not provided.	Annual information obligations for packaging waste operators (Art. 23). Packaging waste management in waste management plans (Art. 42).	Not provided.	Does not directly regulate waste management, but sets requirements to facilitate it through product design.	Regulates the collection and treatment of electronic waste (Art. 5–8).
	Recovery and Recycling	Not provided.	Requires design for recycling and recovery (Annex II).	Promotes recovery and recycling, encouraging separate collection systems and setting quantitative targets (Arts. 4, 10, 11, Annex II).	Not provided.	Establishment of collection systems and prohibition of incineration and landfill for packaging designed in accordance with the regulation (Arts. 48, 49). Quantitative recycling targets (Arts. 52–54).	Not provided.	Does not set direct quantitative targets but promotes recycled content and recyclability through design requirements (Art. 5–6).	Sets minimum collection (from 45% to 65% or 85%) and recovery targets (Art. 7, 11, Annex V).
